# Prevalence of *Neospora caninum* Exposure in Wild Pigs (*Sus scrofa*) from Oklahoma with Implications of Testing Method on Detection

**DOI:** 10.3390/ani11092487

**Published:** 2021-08-25

**Authors:** Katelyn M. Haydett, Steven T. Peper, Cynthia Reinoso Webb, Hannah S. Tiffin, Alexander N. Wilson-Fallon, Yava L. Jones-Hall, Stephen L. Webb, Steven M. Presley

**Affiliations:** 1 Vector-Borne Zoonoses Laboratory, Texas Tech University, Lubbock, TX 79416, USA; haydettk@msu.edu (K.M.H.); speper@amcdfl.org (S.T.P.); cynthia.reinoso@ttu.edu (C.R.W.); hsg14@psu.edu (H.S.T.); afallon65@gmail.com (A.N.W.-F.); steve.presley@ttu.edu (S.M.P.); 2 Anastasia Mosquito Control District, 120 EOC Drive, St. Augustine, FL 32092, USA; 3 College of Veterinary Medicine, Purdue University, West Lafayette, IN 47906, USA; yavajh@cvm.tamu.edu; 4 Noble Research Institute, LLC, 2510 Sam Noble Parkway, Ardmore, OK 73401, USA

**Keywords:** canine neosporosis, cattle, disease, invasive species, livestock, parasite, wild pig

## Abstract

**Simple Summary:**

*Neospora caninum* causes abortion and other reproductive challenges in livestock and leads to large economic losses every year. Wild pigs were evaluated for their potential role in the transmission of *N. caninum*, the parasite that causes neosporosis in cattle and other animals. Three assays were used to identify past or current exposure to *N. caninum* in wild pigs and histology was performed to determine if there was a pathology consistent with a *N. caninum* infection in the tissues that were evaluated. The following assays reported positive results: Kit A—67.8%, Kit B—12.5%, Indirect Fluorescent Antibody Test—84.1%, and Histology—0%. Importantly, the assays used in this study were not congruent with all duplicate samples or between test types and demonstrate the need for a more reliable test to identify *N. caninum* infections in wild pigs to better assess their potential role in disease transmission.

**Abstract:**

*Neospora caninum* is a protozoan parasite, reported as a leading cause of cattle abortions and reproductive failure worldwide, costing the cattle industry approximately $1.3 billion annually. With wild pig (*Sus scrofa*) populations estimated at over six million in the United States, contact between wild pigs and livestock is inevitable, mainly because of the widespread geographic co-occurrence of the two species. As a known reservoir for numerous fungal, bacterial, viral and parasitic diseases, wild pigs are of particular importance for human and veterinary health relative to the prevention of infectious diseases. The seroprevalence of *N. caninum* in wild pig populations was previously documented in the United States, raising the question as to their exposure point of prevalence. This research screened 116 individual wild pigs for *N. caninum* using a variety of available assays. Using two different commercially available ELISA test kits, seroprevalence ranged from 12.5% to 67.8%. The Indirect Fluorescent Antibody Test resulted in our highest percent seroprevalence for these samples, at 84.1%. However, none of our samples showed any presence of *N. caninum* or associated pathologies via histological evaluation of representative tissues. Importantly, the assays used in this study were not congruent with all duplicate samples or between the test types used. The implications of these non-congruent results demonstrates that currently available testing assays produce variable results, underscoring the need for more reliable testing kits and a standardized methodology when assessing disease prevalence in wildlife, particularly for *N. caninum* in wild pigs, which impacts prevalence and comparability across studies.

## 1. Introduction

Wild pigs (*Sus scrofa*) play an instrumental role in disease transmission to humans, livestock and wildlife [1,2]. To make matters worse, states that have the highest cattle populations in the United States also have the highest wild pig populations. For example, Texas is ranked first in the United States for cattle production, with 12.5 million head, and first for wild pig populations, with an estimated population of 2.6 million. Oklahoma is ranked fifth for cattle production, with 5.1 million head and fourth for wild pig population, which is approximated at 500,000 [3,4]. The total wild pig population within the United States is estimated to be 6.3 million, with 99% of the total population found within ten states: Alabama, Arkansas, California, Florida, Georgia, Louisiana, Mississippi, Oklahoma, South Carolina and Texas [3]. As the range of wild pigs continues to expand in the United States, contact between wild and domestic animals is becoming more frequent and, in the case of wild pigs, rapid range expansion and overlap in habitat with livestock poses a significant threat for the potential spread of zoonotic diseases [1].

Diseases that involve both domestic and wild animals are often difficult to control and may result in both social and economic impacts [5]. Neosporosis, caused by the protozoan parasite *Neospora caninum*, is recognized as an economically important disease due to its impact on the livestock industry [6,7] and is considered to be one of the most efficiently transmitted parasites of cattle [8,9,10]. Although *N. caninum* is not zoonotic, infection with *N. caninum* in cattle may result in reproductive failures, resulting in major economic losses [2]. Globally, the annual cost of *N. caninum*-associated abortions averages at $1.298 billion and is estimated to be as high as $2.38 billion [11]. Due to its significant role in cattle abortions, a more in-depth understanding of this parasite and disease is required in order to better understand the domestic and sylvatic lifecycles.

The lifecycle of *N. caninum* consists of three infectious stages: tachyzoites, bradyzoites and oocysts. Tachyzoites rapidly divide within the hosts’ cells and are capable of producing several hundred new parasites each day [6,12]. This asexual reproduction typically occurs within the kidneys, liver, and spleen [13]; however, tachyzoites have been identified in other tissues, such as the brain, heart, muscle and placenta [14,15,16,17]. Infected animals that are pregnant may transmit *N. caninum* tachyzoites across the placenta, infecting the embryo(s) or fetus(es) [12]. Tachyzoites become encysted and differentiate into bradyzoites [9], which are commonly referred to as tissue cysts.

Different parasitic stages are present in intermediate and definitive hosts. Intermediate hosts have tachyzoites and bradyzoites circulating in their blood and located in tissues. Definitive hosts consume infected intermediate host tissues that harbor the tachyzoite or bradyzoite stage and then they release the oocysts in their feces. Once in the environment, *N. caninum* oocysts sporulate and remain environmentally resistant in soil, food, and water, which allows for convenient ingestion by intermediate hosts once again [12,17]. It is hypothesized that all definitive hosts are carnivorous animals because they consume meat on a regular basis and are thus more prone to consume infected tissues, while intermediate hosts tend to be herbivorous [12]. As wild pigs are omnivorous [18], they could potentially serve as either an intermediate or definitive host.

To date, over 139 wild and domestic species have been identified and hypothesized to serve as intermediate hosts after testing for seroprevalence and/or parasitic DNA (reviewed in [19]). However, *N. caninum* has only been successfully isolated from six species, confirming cattle, sheep, dogs, white-tailed deer, bison and water buffalo as intermediate hosts [20,21,22], with cattle being the most well documented [12]. Unfortunately, the seroprevalence of *N. caninum* in wild pigs has not been well studied [23]. Ten studies have focused on *N. caninum* exposure in wild pigs specifically (Table 1), and only two of those reports were on populations within the United States [24,25].

The purpose of this study was to assess the seroprevalence of *N. caninum* from a wild pig population in Oklahoma, to determine if these animals displayed pathological lesions and/or if the *N. caninum* organism could be identified in tissue, and to determine if lesions/organisms correlate with seropositivity, thereby demonstrating their potential to serve as intermediate hosts. Herein, we used three test methods to estimate the seroprevalence of *N. caninum* and further evaluate the consistency of results across testing methods, as well as histology. Ideally, the findings and conclusions of this study will encourage the development of consistent and reliable test assays to determine wild pigs’ (and wildlife in general) exposure to *N. caninum*.

## 2. Materials and Methods

### 2.1. Study Sites

All study sites were within the Cross Timbers and Prairies ecoregions [33]. This ecoregion is characterized by mixed woody vegetation communities consisting of natural openings where the predominant species are oaks (*Quercus* spp.), elms (*Ulmus* spp.) and hickories (*Carya* spp.). Bottomlands also are common and consist of oaks, ashes (*Fraxinus* spp.), elms, hackberries (*Celtis* spp.), pecan (*Carya illinoinensis*) and Osage orange (*Maclura pomifera*). Herbaceous vegetation such as bluestems (*Andropogon* and *Schizachrium* spp.), switchgrass (*Panicum virgatum*), Indiangrass (*Sorghastrum nutans*) and various forbs are predominant in openings [33]. Study sites included six ranches in southern Oklahoma: Coffey, Kuehny, Oswalt, Red River, Strate and Ljungdahl (Table 2, Table S1). One additional location, Texas, indicates samples that were originally trapped and collared in Oklahoma on Red River Ranch, but harvested across the Texas state border (Table 2, Table S1).

### 2.2. Sample Collection

Samples were collected from southcentral Oklahoma (Carter, Garvin and Love counties) in collaboration with the Noble Research Institute as part of the wild pig control and eradication efforts. Wild pigs were trapped from 2015 through 2018 using a BoarBuster trap (WW Livestock Systems, Thomas, OK, USA), which allowed for the capture of entire sounders [34,35]. All traps were baited with whole-kernel corn. Six hundred and seventy-three wild pigs were collected as part of the eradication program and, of those, 106 were randomly selected for testing as part of this study. Ten additional samples were included from Kuehny Ranch (Garvin County, OK, USA) because of a recent case of abortion in a beef cow that was attributed to *N. caninum* (B. Kuehny, personal communication [36]). These 116 samples included both males and females. County, trap site, sex, and age for each sample are listed in Table S1.

Blood and tissue samples were opportunistically collected post euthanasia. Whole blood was collected via heart stick cardiac puncture into serum separator tubes (BD Vacutainer, Franklin Lakes, NJ, USA). Samples were allowed to clot for at least 30 min and then centrifuged in the field at 1000× *g* for 20 min. Samples were stored on ice for transport and then stored in a freezer (≤−20 °C) until testing at the Vector-borne Zoonoses Laboratory at Texas Tech University (Lubbock, TX, USA). The samples utilized for this study were also evaluated as part of a previously published work focused on *Brucella* spp., *Francisella tularensis* and Pseudorabies virus [37,38].

### 2.3. Testing Procedures

Initially, samples were tested using a commercially available qualitative porcine *Neospora caninum* ELISA (enzyme-linked immunosorbent assay) kit (MyBioSource, Cat. No. MBS078173; hereafter referred to as “Kit A”). However, we found inconsistencies between plates and duplicate samples (see Supplementary Information, Table S2). For this reason, several other testing platforms were utilized for comparison. A second commercially available ELISA kit (VMRD, Cat. No. 280-2; hereafter referred to as “Kit B”), Indirect Fluorescent Antibody Test (IFAT), and histology examination were also conducted on select samples. Manufacturer’s instructions were followed on all commercial ELISA kit testing. Samples reported as positive indicate the presence of antibodies against *N. caninum*. Positive results do not necessarily support a current infection, but rather serve as an indication of exposure. Negative results indicate that the antibodies against *N. caninum* were not detected in the serum sample. Negative results indicate that either the animal was not exposed to *N. caninum* or was tested during the window period before the development of detectable antibodies.

### 2.4. Indirect Fluorescent Antibody Test (IFAT)

The wild pig serum samples were tested via IFAT using reagents from Veterinary Medical Research and Development (VMRD). *Neospora caninum* FA Substrate Slide (Cat. No. SLD-IFA-NC) containing NC-1 tachyzoites were inoculated with 10 µL of serum from wild pigs. Slides were incubated at 37 °C for 30 min and rinsed using phosphate-buffered saline (PBS). The sample wells were inoculated with 10 µL of anti-porcine IgG FITC Conjugate Affinity Purified (Cat. No. CJ-F-PORG-AP-10 mL). As controls, we used VMRD *Neospora caninum* FA Positive and Negative Control (bovine) (Cat. No. PC-IFA-NC-BOV and NC-IFA-NC-BOV, respectively) to inoculate the substrate slide. These wells were labeled with Anti-Bovine IgG1,2 (H&L) FITC Conjugate Affinity Purified (Cat. No. CJ-F-BOVG-AP-10 ML). A total of 5 µL of Fluoromount G™ (SouthernBiotech; Cat. No. 0100-01) was used as mounting fluid and a cover slip was placed on the slide. Slides were read at 400× using a fluorescent microscope.

To ensure that no non-specific binding occurred that would yield to false positives, *Neospora caninum* FA substrate slides were inoculated with the following mixtures: bovine positive control plus porcine FITC conjugate; sterile distilled water plus porcine FITC conjugate; negative test serum plus bovine FITC conjugate. All these combinations were treated the same as test samples and all were negative, demonstrating that there was no non-specific binding of the conjugates to the substrate or serum.

### 2.5. Tissue Collection and Processing

Necropsy was performed on the euthanized animals and tissue sections of heart, tongue, liver, kidney and spleen were collected from select female and male wild pigs. The sections were submerged in 10% neutral buffered formalin for at least 48 h to allow for proper fixation. Tissue processing was carried out at the Department of Pathology at Texas Tech Health Sciences Center (Lubbock, TX, USA). Fixed sections of the tissues were embedded in paraffin, cut into 6-μm sections, and stained with hematoxylin and eosin (H&E) for histological analysis via light microscopy at Purdue University College of Veterinary Medicine (West Lafayette, IN, USA).

## 3. Results

Samples were tested using Kit A (88), Kit B (89), IFAT (113), and/or histology (26). Fifty percent (58/116) of the individual wild pigs had all four tests performed, 47.4% (55/116) had three of the four tests, and 2.6% (3/116) had two of the tests performed (Table 2).

Of the 58 samples that were tested with all three antibody tests, only 6.9% (4/58) agreed between all three tests. However, two of those individuals had duplicates for a specific test that were not congruent, so a more conservative estimate would be that only 3.5% (2/58) agreed across all three tests.

### 3.1. Kit A

Eighty-eight samples were screened for *N. caninum* antibodies using Kit A. Samples were run in duplicate on each plate. Due to the inconsistency of duplicate samples, several samples were rerun on multiple plates for comparison. Raw data from duplicate samples and multiple plates are reported in Table S2. Optical densities from duplicates were averaged, resulting in a positive or negative assignment (Table 2). Seventy-five percent (66/88) of the samples tested positive. Thirty-three percent (29/88) of duplicate samples had results that were not congruent with each other (one reporting as positive and one as negative). If these non-congruent samples are excluded from analysis, then the positive rate falls to 67.8% (40/59).

### 3.2. Kit B

Eighty-nine samples were screened for *N. caninum* antibodies using Kit B. Samples were run in duplicate on each plate and the OD value was converted to a percent inhibition as per the manufacturer’s instructions (Table S3). Duplicate percent inhibitions were averaged, and the results reported in Table 2. Samples tested positive in 16.9% (15/89) of the samples tested. Ten percent (9/89) of duplicate samples had results that were not congruent with each other (one reporting as positive and one as negative), resulting in a conservative positive rate of 12.5% (10/80) when excluding non-congruent samples from analysis.

### 3.3. IFAT

One hundred and thirteen samples were screened for *N. caninum* antibodies using IFAT, with 95 samples (84.1%) testing positive (Table 2).

### 3.4. Histology

Sections of heart, tongue, spleen and liver from 27 animals were histologically evaluated by a board-certified veterinary pathologist (Dr. Yava Jones-Hall) for the presence of *N. caninum* cysts and pathologic lesions. *Neospora* cysts were not identified (Table 2); however, incidental lesions were noted in several sections from most animals (Table S4). Cardiac lesions ranging from minimal to mild, mostly focal nonsuppurative inflammation (11%), evidence of mild arterial hypertrophy (26%), mild, myocardial fibrosis (1 animal) and an intact *Sarcocyst* cyst, without evidence of a tissue response (1 animal), were noted. In the tongue sections, 40% of the animals presented intramuscular, intact *Sarcocyst* cysts and 19% of animals exhibited focal mild, nonsuppurative inflammation and/or focal cartilaginous metaplasia of the muscle. Evaluation of the liver revealed that all but one animal had mild vacuolar hepatopathy and few (19%) had mild, focal, mixed inflammation. Twenty-six percent of the animals had from minimal to mild, nonsuppurative inflammation present focally or multifocally in the cortex of the kidneys. The only notable lesion in the spleen was a subjectively mild increase in macrophages in the parenchyma in most animals (93%).

## 4. Discussion

A large degree of uncertainty exists in the world of wildlife disease serology; however, the information that is collected, such as those collected in this study, is vital for the advancement of disease surveillance and control. When it comes to *N. caninum*, little is known about the exact roles that wildlife play in introducing and maintaining infection. Excluding experimental infection studies, the highest reported seroprevalence of *N. caninum* in pigs to date occurred in Senegal, where 60 sows were tested over 3 years and had a 58.3% seroprevalence [39]. The second highest seroprevalence was observed in a wild pig population in Michigan, United States, with 57.1% of samples testing positive for antibodies to *N. caninum* [25]. Cerqueira-Cézar, and others [25] also reported 0% seroprevalence in wild pig populations in California, Nevada, New York, Pennsylvania, Utah, and Virginia.

While this seroprevalence assessment did not determine the exact role wild pigs play in transmitting this parasite, it did conclude that wild pigs may serve as an intermediate host, given their exposure to *N. caninum,* as found in this study. Proper action must be taken to prevent the potential spread of this disease through wild pigs. This study also evaluated the lack of congruency within and between evaluation assays for *N. caninum*. Of the previous reported studies of pigs and *N. caninum*, only seven used a secondary confirmation test to validate the results. The lack of confirmatory testing is not uncommon in seroprevalence studies, especially in nondomestic animals, in which no methods are validated by a gold standard. Due to this lack of further validation and the wide variety of detection methods currently used by various labs around the world, comparability between studies is difficult and should be interpreted cautiously [40]. Seroprevalence testing in wildlife disease research faces numerous problems when it comes to diagnostic test kit validation and determination of test specificity and selectivity. Identifying reliable and consistent diagnostic assays is critical for any effective disease surveillance program.

A wide variety of tests are currently available for the detection of *N. caninum* antibodies in serum samples. Some of these methods include *Neospora* agglutinating testing (NAT), IFAT, and a variety of ELISAs. Due to their specificity and sensitivity, IFAT and ELISA are most commonly used in the literature. *Neospora* agglutinating testing is known to have similar specificity and sensitivity to IFAT, but it does not require species-specific antibodies, which is helpful when studying wildlife diseases [41]. The polymerase chain reaction (PCR) is another option for the detection of different organisms, given its having high specificity and sensitivity. However, the sample must be collected during active infection and while viremia is high enough to be detected.

In this study, Kit A, and to a lesser degree, Kit B showed high rates of inconsistency between duplicate samples, as one sample would report as negative and its duplicate would report as positive on the same plate. Kit A was species-specific when testing porcine samples. While Kit B is specified for use in cattle, the overall design does allow for serum samples from various animals to be tested [42]. Due to this design principle, the same kit (Kit B) has been used in over 42 species (including wild pigs) as previously outlined in Haydett [19].

Kit A differed drastically in the observed seroprevalences compared to studies previously conducted in the same area in Oklahoma. Kit A resulted in an observed seroprevalence (75.0/67.8%) over four times greater than the previously reported average seroprevalence (15.9%, [25]), while Kit B was more consistent at 16.9/12.5% seroprevalence.

IFAT is often used as a confirmatory test [29] because it is very specific, no cross reactions exist with similar parasites [43], and it can be used on multiple species [27]. In this study, IFAT results had the highest percent seroprevalence at 84.1% (95/113), over five times that previously reported for the area [25]. The downsides to using IFAT routinely include the increased cost, need for species-specific conjugates, and the additional testing time, and specialized training required to perform this test [43,44].

When considering possible causes for this lack of congruency among our samples and tests, we considered the sample quality. However, the sample quality in this study was confirmed through various methods. While, in some cases, different aliquots were used, all samples from an individual were collected at the same time, spun down, and mixed before serum aliquots were removed. The accuracy of equipment such as the plate washer and plate reader were confirmed, as other studies used the same equipment and did not observe any differences in the congruency among duplicate samples. These same individual wild pigs were also used as part of a different disease surveillance project that utilized the same serum samples for antibody testing and did not have any duplicate sample incongruencies [38].

In an effort to validate our testing procedures, serum from wild pigs considered positive for *N. caninum* were obtained to serve as species-specific positive controls with Kit B. Even though these samples had been previously tested and considered positive via NAT [25], they did not test positive with Kit B. This lack of congruency raises yet another limitation because even when species-specific controls can be obtained, they are not necessarily compatible with the method being used. This lack of consistency remains a challenge in wildlife disease research because, without previously tested samples that have documentation of their positivity and negativity, validation of test kits is extremely difficult. Unfortunately, due to the limited quantity of the positive controls that we were able to obtain from previous studies, we were not able to validate Kit A or IFAT with these controls.

Due to their increasing population, generalist diet, and adaptability to inhabit a diverse range of habitats, wild pigs can serve as excellent surveillance tools for the presence of disease [45]. For these reasons, future research should focus on detecting the presence of *N. caninum* DNA in wild pig tissues, isolating the organism, detecting *N. caninum* oocytes in wild pig feces, and confirming their role as an intermediate or potential definitive host. In doing so, the current test kits and detection methodology can be validated for use on wild pigs and provide a way to assess the presence of infectious diseases in the environment. Research should be continued to further assess and develop detection methods for seroprevalence in wildlife so that *N. caninum* disease risk may be better assessed and mitigated.

## 5. Conclusions

This study documented the presence of *N. caninum* antibodies in wild pig populations from southcentral Oklahoma. With the lack of microscopy evidence in the tissue samples, histopathology could not be correlated with seropositivity in this study. Overall, Kit A reported a 67.8% seroprevalence, Kit B a 12.5% seroprevalence, and IFAT an 84.1% seroprevalence. Unfortunately, there were discrepancies between duplicate sample results within and among test assays. These results reveal the lack of consistent and reliable test assays available for screening *N. caninum* exposure in wild pigs. The lack of consistent and reliable wild pig *N. caninum* positive controls is another issue that needs to be addressed in future studies. Species- and pathogen-specific positive controls are needed for the effective development and validation of any new assay. The lack of positive controls in wildlife studies is an ongoing issue and has the potential to negatively impact the reported prevalence rate and comparability across studies. Knowing that contact between wild pigs and livestock is inevitable, it is imperative that studies are able to properly evaluate disease prevalence, such as *N. caninum*, in wild pig populations, because the uncontrolled and unmonitored presence of disease can have catastrophic financial consequences. Herein, we identify the need for the development of consistent and reliable *N. caninum* assays for use with wild pigs as well as the development or identification of reliable positive controls for use in off-label assays.

## Figures and Tables

**Table 1:** Summary of published studies examining *Neospora caninum* in wild pigs.

Year	Location	Seroprevalence		Method	Citation
		(*n*)	(%)		
2006	Czech Republic	565	18.1/10.3	ELISA/IFAT	[26]
2007	Spain	298	0.6/0.3	ELISA/IFAT	[27]
2011	Germany	1	0	PCR	[28]
2013	United States	467	15.8	ELISA	[24]
2015	Turkey	12	0	ELISA	[29]
2015	Grenada	185	0	ELISA	[30]
2015	Greece	94	1.1	IFAT	[31]
2016	Brazil	83	10.8	IFAT	[22]
2016	Slovakia	113	33.6/20.4	ELISA/PCR	[32]
2016	United States	1059	15.0	NAT	[25]

ELISA = enzyme-linked immunosorbent assay; IFAT = Indirect Fluorescent Antibody Test; PCR = polymerase chain reaction; NAT = neospora agglutinating testing.

**Table 2:** Serological and histological results from 116 wild pigs captured from southcentral. Oklahoma, 2015 through 2018. Positive samples reported with total samples tested in parentheses.

	Ranch	Kit A	Kit B	IFAT	Histology
Carter	Ljungdahl	1 (1)	0 (1)	1 (1)	-
Cook	Texas	0 ^a^ (1)	0 (1)	0 (1)	-
Garvin	Kuehny	0 ^b^ (10)	0 (6)	10 (10)	-
Love	Coffey	14 ^c^ (21)	3 ^e^ (15)	17 (19)	-
	Oswalt	2 (2)	0 (2)	2 (2)	-
	RedRiver	21 ^d^ (51)	7 ^f^ (63)	64 (78)	0 (26)
	Strate	2 (2)	0 (1)	2 (2)	-

^a^ 1 Sample was inconclusive, ^b^ 10 samples were inconclusive, ^c^ 4 samples were inconclusive, ^d^ 14 samples were inconclusive, ^e^ 1 sample was inconclusive, ^f^ 8 samples were inconclusive.

## Data Availability

The data used herein may be made available upon reasonable requests by contacting the corresponding author (S.L.W.).

## Supplementary Materials

The following are available online at https://www.mdpi.com/article/10.3390/ani11092487/s1, Table S1: Serological and histological results from 116 wild pigs captured from southcentral Oklahoma, 2015–2018, Table S2: OD values for each sample and its duplicate for Kit A. OD values greater to or equal to the cutoff value represents a positive sample, Table S3: Percent inhibition values for each sample and its duplicate for Kit B. Percent inhibition values greater to or equal to 30 represents a positive sample. Percent inhibition was calculated per the manufacturer’s instructions: % I = 100[1 − (Sample OD/Average Negative Kit Control OD)], Table S4: Histological findings from 26 wild pigs and one fetus via light microscopy.
