# Supplementary material for: Prevalence of Neospora caninum Exposure in Wild Pigs (Sus scrofa) from Oklahoma with Implications of Testing Method on Detection
Source: Animals (Basel). 2021 Aug 25;11(9):2487. doi: 10.3390/ani11092487 (PMC8465085; doi:10.3390/ani11092487)
Supplement: Supplementary file 1 [file animals-11-02487-s001.zip › animals-1323157-supplementary.pdf]

Supplementary Materials:

Table S1. Serological and histological results from 116 wild pigs captured from southcentral Oklahoma, 2015 through 2018.

Table S2. OD values for each sample and its duplicate for Kit A. OD values greater to or equal to the cutoff value represents a positive sample.

Table S3. Percent inhibition values for each sample and its duplicate for Kit B. Percent inhibition values greater to or equal to 30 represents a positive sample. Percent inhibition was calculated per the manufacturer's instructions: % I = 100[1-(Sample OD/Average Negative Kit Control OD)].

Table S4. Histological findings from 26 wild pigs and one fetus via light microscopy.

Table S1. Serological and histological results from 116 wild pigs captured from southcentral Oklahoma, 2015 through 2018.

Sample ID[a]	County	Ranch	Sex	Age	Kit A	Kit B	IFAT	Histology
151068	Love	Oswalt	Male	Juv	Pos	Neg	Pos	--
151077	Love	Oswalt	Female	Sub	Pos	Neg	Pos	--
151078	Love	RedRiver	Female	Ad	Pos	Neg	Neg	--
151079	Love	RedRiver	Female	Ad	Pos	Neg	Pos	--
151081	Love	RedRiver	Male	Juv	Pos	Neg	Pos	--
151084	Love	RedRiver	Female	Juv	Incon.	Neg	Pos	--
151085	Love	RedRiver	Male	Juv	Pos*	Neg	--	--
151089	Love	RedRiver	Female	Juv	--	Neg	Pos	--
151093	Love	RedRiver	Male	Juv	Pos	Neg	Pos	--
160005	Love	RedRiver	Female	Ad	Incon.	--	Pos	--
160009	Love	RedRiver	Female	Sub	Pos	Neg	Neg	--
160013	Love	RedRiver	Female	Ad	Incon.	Neg	Neg	--
160016	Love	RedRiver	Female	Ad	Incon.	Neg	Neg	--
160018	Love	RedRiver	Female	Ad	--	Neg	Pos	--
160019	Love	RedRiver	Male	Ad	Pos	Neg	Pos	--
160021	Love	RedRiver	Male	Ad	Incon.	Neg	Pos	--
160023	Love	RedRiver	Female	Sub	Incon.	Neg	Pos	--
160024	Love	RedRiver	Female	Sub	Incon.	Neg	Pos	--
160025	Love	RedRiver	Female	Ad	Incon.	Neg	Pos	--
160067	Love	Coffey	Male	Juv	Pos	Neg	Pos	--
160073	Love	Coffey	Female	Ad	Pos	Neg	Pos	--
160074	Love	Coffey	Female	Sub	Pos	Neg	--	--
160075	Love	Coffey	Female	Ad	Neg	Neg	Pos	--
160077	Love	Coffey	Female	Ad	Pos	--	Neg	--
160095	Love	Coffey	Male	Ad	Pos	--	Pos	--
160099	Love	Coffey	--	--	Incon.	Pos	Neg	--
160108	Love	Coffey	--	--	Incon.	Pos	--	--
160109	Love	Coffey	--	--	Pos	Neg	Pos	--
160113	Love	Coffey	--	--	Pos	Neg	Pos	--
160115	Love	Coffey	--	--	Incon.	Incon.	Pos	--
160118	Love	Coffey	Female	Juv	Pos	Neg	Pos	--
160121	Love	Coffey	Female	--	Incon.	Pos	Pos	--
160123	Love	Coffey	Male	--	Pos	Neg	Pos	--
160127	Love	Coffey	Male	Ad	Pos	Neg	Pos	--
160137	Garvin	Kuehny	Male	--	Incon.	--	Pos	--
160138	Garvin	Kuehny	Female	--	Incon.	Neg	Pos	--
160139	Garvin	Kuehny	Male	--	Incon.	--	Pos	--
160140	Garvin	Kuehny	Male	--	Incon.	--	Pos	--
160141	Garvin	Kuehny	Female	--	Incon.	Neg	Pos	--
160142	Garvin	Kuehny	Female	--	Incon.	Neg	Pos	--
160143	Garvin	Kuehny	Female	--	Incon.	Neg	Pos	--
160144	Garvin	Kuehny	Male	--	Incon.	Neg	Pos	--
160145	Garvin	Kuehny	Female	--	Incon.	--	Pos	--
160146	Garvin	Kuehny	Male	--	Incon.	Neg	Pos	--
160150	Carter	Ljungdahl	Female	--	Pos	Neg	Pos	--
160899	Love	Strate	Male	Ad	Pos	--	Pos	--
160900	Love	Strate	Male	Juv	Pos	Neg	Pos	--
160904	Love	RedRiver	Female	--	Neg	Neg	Pos	--
160906	Love	RedRiver	Female	Ad	Pos	--	Pos	--
160910	Love	RedRiver	Female	Ad	Pos	Neg	Pos	--
160912	Love	RedRiver	Male	Ad	Pos	Neg	Pos	--
160916	Love	RedRiver	Male	Sub	Pos	--	Pos	--
160919[b]	Love	RedRiver	Male	Ad	Pos	Pos	Pos	--
160921	Love	RedRiver	Male	Juv	Pos	--	Pos	--
160922	Love	RedRiver	Male	Juv	Neg	Neg	Pos	--
160925	Love	RedRiver	Female	Juv	Pos	Incon.	Pos	--
160929	Love	RedRiver	Male	Juv	Neg	Neg	Pos	--
170001	Love	RedRiver	Male	Ad	Pos	Neg	Pos	--
170002	Love	RedRiver	Female	Ad	Pos	--	Neg	--
170004	Love	RedRiver	Female	Sub	Pos	Neg	Pos	--
170005	Love	RedRiver	Female	Ad	Pos	Neg	Pos	--

170006	Love	RedRiver	Female	Sub	Neg	--	Neg	--
170007	Love	RedRiver	Female	Sub	Pos	Neg	Neg	--
170008	Love	RedRiver	Female	Ad	Pos	Neg	Pos	--
170011	Love	RedRiver	Male	--	Neg	--	Pos	--
170018	Love	RedRiver	Female	--	Neg	Pos	Pos	--
170022	Love	RedRiver	Female	--	Incon.	Neg	Pos	--
170025	Love	RedRiver	Male	Pgl	Incon.	--	Pos	--
170034	Love	RedRiver	Male	Pgl	Neg	Incon.	Neg	--
170035[c]	Love	RedRiver	Female	--	Neg	Neg	Neg	--
170037	Love	Coffey	Female	Ad	Pos	Neg	Pos	--
170038	Cooke	Texas[d]	Female	Ad	Incon.	Neg	Neg	--
170040	Love	RedRiver	Female	Ad	Pos	--	Pos	--
170042	Love	RedRiver	Female	Ad	Neg	--	Neg	--
170045	Love	RedRiver	Female	Ad	Incon.	Neg	Pos	--
170046	Love	RedRiver	Female	Ad	Neg	--	Pos	--
170049	Love	RedRiver	Female	--	Neg	Pos	Neg	--
170053	Love	RedRiver	Female	--	Incon.	Pos	Neg	--
170055	Love	RedRiver	Male	--	Incon.	Pos	Neg	--
170057	Love	RedRiver	Female	Pgl	Incon.	--	Pos	--
170059	Love	RedRiver	Female	Pgl	Neg	--	Pos	--
170062	Love	RedRiver	Male	Pgl	Neg	--	Pos	--
170067	Love	RedRiver	Female	Ad	Neg	--	Neg	--
170072	Love	RedRiver	Male	Ad	Neg	Neg	Pos	--
170095	Love	Coffey	Female	Sub	Neg	--	Pos	--
170098	Love	Coffey	Female	Ad	Neg	--	Pos	--
170125	Love	RedRiver	Female	Ad	Neg	--	Pos	--
170145	Love	Coffey	Female	--	Pos	--	Pos	--
170146	Love	Coffey	Female	--	Pos	Neg	Pos	--
170147	Love	Coffey	Female	Ad	Pos	--	Pos	--
180501	Love	RedRiver	Female	--	--	Neg	Pos	Neg
180502	Love	RedRiver	Male	--	--	Neg	Pos	Neg
180503	Love	RedRiver	Female	--	--	Pos	Pos	Neg
180504	Love	RedRiver	Male	--	--	Incon.	Pos	Neg
180505	Love	RedRiver	Female	--	--	Neg	Pos	Neg
180506	Love	RedRiver	Male	--	--	Neg	Pos	Neg
180507	Love	RedRiver	Female	--	--	Neg	Pos	Neg
180508	Love	RedRiver	Male	--	--	Neg	Pos	Neg
180509	Love	RedRiver	Male	--	--	Incon.	Pos	Neg
180510	Love	RedRiver	Male	--	--	Neg	Pos	Neg
180511	Love	RedRiver	Female	--	--	Neg	Pos	Neg
180512	Love	RedRiver	Female	--	--	Incon.	Pos	Neg
180513	Love	RedRiver	Male	--	--	Neg	Pos	Neg
180514	Love	RedRiver	Male	--	--	Neg	Pos	Neg
180515	Love	RedRiver	Male	--	--	Neg	Pos	Neg
180516	Love	RedRiver	Male	--	--	Neg	Pos	Neg
180517	Love	RedRiver	Male	--	--	Pos	Pos	Neg
180518	Love	RedRiver	Female	--	--	Incon.	Pos	Neg
180519	Love	RedRiver	Female	--	--	Incon.	Pos	Neg
180520	Love	RedRiver	Female	--	--	Neg	Pos	Neg
180521	Love	RedRiver	Female	--	--	Neg	Pos	Neg
180522	Love	RedRiver	Female	--	--	Neg	Pos	Neg
180523	Love	RedRiver	Male	--	--	Neg	Pos	Neg
180524	Love	RedRiver	Male	--	--	Neg	Pos	Neg
180525	Love	RedRiver	Female	--	--	Incon.	Pos	Neg
180526	Love	RedRiver	Female	--	--	Neg	Pos	Neg

Pos = Positive; Neg = Negative; Incon. = Inconclusive where duplicate samples were not congru-ent (i.e., one tested positive while the other tested negative); * Sample had limited quantity and was not run in duplicate; a First two digits of the ID represent the year the animal was sampled; b Sample that was positive for all three serological assays; c Sample that was negative for all three serological assays; d Originally captured in RedRiver (Love County)

Table S2. OD values for each sample and its duplicate for Kit A. OD values greater to or equal to the cutoff value represents a positive sample.

Sample ID	First Run			Second Run			Third Run			Fourth Run			Overall OD Ave
	OD1	OD2	OD Ave	OD1	OD2	OD Ave	OD1	OD2	OD Ave	OD1	OD2	OD Ave	
Cut-Off			0.223			0.241			0.217			0.212	0.22325
Neg	0.075	0.07	0.0725	0.091	0.091	0.091	0.067	0.067	0.067	0.061	0.063	0.062	0.07313
Pos	1.896	1.62	1.758	3.129	3.096	3.1125	2.526	2.562	2.544	2.805	2.644	2.7245	2.53475
151068	0.522	0.536	0.529										0.529
151077	0.493	0.521	0.507				0.961	0.983	0.972				0.7395
151078	0.488	0.517	0.5025										0.5025
151079	0.442	0.447	0.4445										0.4445
151081	0.494	0.495	0.4945										0.4945
151084*	0.411	0.405	0.408				0.049	0.047	0.048	0.918[‡]		0.918	0.458
151085										1.535[‡]		1.535	1.535
151093	0.528	0.559	0.5435				2.294	2.275	2.2845				1.414
160005*										0.55	0.052	0.301	0.301
160009	0.518	0.51	0.514										0.514
160013*	0.053	0.481	0.267										0.267
160016*	0.054	0.517	0.2855				0.05	0.048	0.049				0.16725
160019	0.517	0.527	0.522										0.522
160021*	0.059	0.463	0.261				0.623	0.603	0.613				0.437
160023*										0.686	0.084	0.385	0.385
160024*										0.508	0.054	0.281	0.281
160025*										0.624	0.052	0.338	0.338
160067	0.559	0.566	0.5625										0.5625
160073										1.833	1.354	1.5935	1.5935
160074										0.422	0.338	0.38	0.38
160075										0.055	0.049	0.052	0.052
160077										0.721	1.344	1.0325	1.0325
160095	0.518	0.551	0.5345										0.5345
160099*	0.553	0.058	0.3055										0.3055
160108*	0.559	0.056	0.3075										0.3075
160109	0.555	0.571	0.563				1.103	1.258	1.1805				0.87175
160113	0.563	0.532	0.5475										0.5475
160115*	0.543	0.055	0.299										0.299
160118	0.475	0.511	0.493				0.636	0.845	0.7405				0.61675
160121*	0.431	0.067	0.249										0.249
160123	0.644	0.575	0.6095										0.6095
160127	0.513	0.562	0.5375				0.638	0.642	0.64				0.58875
160137*	0.453	0.48	0.4665	0.651	0.654	0.6525							0.5595
160138*	0.056	0.431	0.2435	0.063	0.698	0.3805	0.579	0.66	0.6195				0.4145
160139*	0.536	0.517	0.5265	0.057	0.642	0.3495							0.438
160140*	0.505	0.055	0.28	0.066	0.615	0.3405	0.046	0.047	0.0465				0.22233
160141*	0.054	0.056	0.055	0.055	0.607	0.331							0.193
160142*	0.057	0.598	0.3275	0.59	0.637	0.6135							0.4705
160143*	0.526	0.052	0.289	0.626	0.612	0.619							0.454

160144*	0.082	0.499	0.2905	0.678	0.492	0.585	1.353	1.288	1.3205				0.732
160145*	0.506	0.053	0.2795	0.769	0.605	0.687							0.48325
160146*	0.055	0.411	0.233	0.655	0.709	0.682	0.554	0.747	0.6505				0.52183
160150	0.524	0.459	0.4915				0.85	0.898	0.874				0.68275
160899							0.752	0.901	0.8265				0.8265
160900							1.071	1.036	1.0535				1.0535
160904							0.053	0.071	0.062				0.062
160906							1.743	1.627	1.685				1.685
160910							1.073	1.25	1.1615				1.1615
160912							1.271	1.185	1.228				1.228
160916							2.071	1.978	2.0245				2.0245
160919							0.902	0.797	0.8495				0.8495
160921							1.296	0.83	1.063				1.063
160922							0.055	0.056	0.0555				0.0555
160925							0.654	0.583	0.6185				0.6185
160929							0.047	0.046	0.0465				0.0465
170001							0.538	0.529	0.5335				0.5335
170002							0.63	0.625	0.6275				0.6275
170004										1.849	1.475	1.662	1.662
170005										0.498	0.458	0.478	0.478
170006							0.054	0.058	0.056				0.056
170007							0.667	0.543	0.605				0.605
170008										0.68	0.661	0.6705	0.6705
170011							0.053	0.058	0.0555				0.0555
170018							0.055	0.054	0.0545				0.0545
170022*							1.098	0.051	0.5745				0.5745
170025*							0.915	0.046	0.4805				0.4805
170034							0.046	0.046	0.046				0.046
170035							0.047	0.047	0.047				0.047
170037										0.75	1.02	0.885	0.885
170038*										0.055	0.5	0.2775	0.2775
170040							0.765	0.865	0.815				0.815
170042							0.06	0.048	0.054				0.054
170045*										0.052	1.293	0.6725	0.6725
170046										0.053	0.052	0.0525	0.0525
170049							0.05	0.049	0.0495				0.0495
170053*							0.439	0.047	0.243				0.243
170055*							0.046	0.582	0.314				0.314
170057*							0.046	0.736	0.391				0.391
170059							0.047	0.05	0.0485				0.0485
170062							0.051	0.049	0.05				0.05
170067							0.116	0.048	0.082				0.082
170072							0.048	0.047	0.0475				0.0475
170095										0.064	0.062	0.063	0.063
170098										0.057	0.055	0.056	0.056
170125										0.062	0.058	0.06	0.06
170145										0.986	0.761	0.8735	0.8735

170146	0.479	0.554	0.5165	0.5165
170147	1.051	1.555	1.303	1.303

* Represents samples that did not have congruent replicate samples

‡ Sample had limited quantity and was not run in duplicate

Table S3. Percent inhibition (% I) values for each sample and its duplicate for Kit B. Percent inhibition values greater to or equal to 30 represents a positive sample. Percent inhibition was calculated per the manufacturer's instructions: % I = 100[1-(Sample OD/Average Negative Kit Control OD)].

Sample ID	First Run			Second Run		
	% Inhibition 1	% Inhibition 2	% Inhibition Ave	% Inhibition 1	% Inhibition 2	% Inhibition Ave
151068				24.0706886	24.19256551	24.13162706
151077				7.739183425	10.05484461	8.897014016
151078				-20.90188909	-20.53625838	-20.71907374
151079				0.548446069	-3.717245582	-1.584399756
151081				1.889092017	1.279707495	1.584399756
151084				-18.2205972	-10.17672151	-14.19865935
151085	12.31964484	3.995560488	8.157602664			
151089	1.553829079	-10.76581576	-4.605993341			
151093				-4.082876295	-8.104814138	-6.093845216
160009				-7.373552712	-3.229737965	-5.301645338
160013	17.31409545	18.09100999	17.70255272			
160016				9.56733699	2.010968921	5.789152956
160018				7.61730652	5.789152956	6.703229738
160019				-11.15173675	-20.78001219	-15.96587447
160021				-1.767215113	-9.932967703	-5.850091408
160023	19.53385128	15.53829079	17.53607103			
160024	4.772475028	5.549389567	5.160932297			
160025	0.998890122	-4.328523862	-1.66481687			
160067				2.985984156	-5.301645338	-1.157830591
160073				0.548446069	-6.398537477	-2.925045704
160074				24.43631932	18.3424741	21.38939671
160075				-25.04570384	-17.85496648	-21.45033516
160099				36.38025594	40.8897014	38.63497867
160108	50.94339623	42.84128746	46.89234184			
160109				13.22364412	20.17062767	16.69713589
160113				28.58013406	21.51127361	25.04570384
160115*	35.8490566	25.08324084	30.46614872			
160118				14.56429007	14.92992078	14.74710542
160121	40.06659267	31.74250832	35.9045505			
160123				26.8738574	16.39244363	21.63315052
160127				8.470444851	6.398537477	7.434491164
160138				21.023766	22.24253504	21.63315052
160141				-6.520414381	-12.7361365	-9.628275442
160142				12.7361365	9.323583181	11.02985984
160143				-7.007921999	-25.41133455	-16.20962828
160144				25.53321146	16.02681292	20.78001219
160146				3.10786106	10.54235222	6.825106642
160150				-10.90798294	-12.00487508	-11.45642901
160900				2.25472273	-1.4015844	0.426569165
160904				10.42047532	8.714198659	9.56733699
160910				-11.15173675	-6.032906764	-8.592321755
160912				24.68007313	3.839122486	14.25959781
160919				36.86776356	34.79585619	35.83180987
160922				17.48933577	26.50822669	21.99878123
160925*	33.62930078	33.62930078	33.62930078	20.78001219	32.3583181	26.56916514
160929				-30.16453382	-34.91773309	-32.54113346
170001				7.982937233	4.448507008	6.215722121
170004				-36.25837904	-31.38330286	-33.82084095
170005				-17.61121268	-15.90493601	-16.75807434
170007				22.24253504	13.58927483	17.91590494
170008				13.71115174	12.12675198	12.91895186
170018				31.50517977	32.84582572	32.17550274
170022	24.97225305	17.64705882	21.30965594			
170034*	30.07769145	18.09100999	24.08435072			
170035				18.82998172	17.85496648	18.3424741
170037				13.95490555	6.276660573	10.11578306
170038	15.76026637	6.548279689	11.15427303			
170045	9.10099889	5.438401776	7.269700333			

170049	32.40843507	39.51165372	35.9600444			
170053	41.95338513	51.83129856	46.89234184			
170055	30.29966704	33.62930078	31.96448391			
170072	14.76137625	18.09100999	16.42619312	11.20976693	9.211986681	10.2108768
170146	2.219755827	9.433962264	5.826859046			
180501	28.85682575	26.8590455	27.85793563			
180502	3.32963374	27.30299667	15.31631521			
180503	40.84350721	32.18645949	36.51498335			
180504*	32.74139845	20.19977802	26.47058824			
180505	16.09322974	3.218645949	9.655937847			
180506	27.96892342	19.86681465	23.91786903			
180507	28.63485017	-15.87125416	6.381798002			
180508	12.65260821	5.660377358	9.156492786			
180509*	40.51054384	29.96670366	35.23862375			
180510	28.30188679	20.86570477	24.58379578			
180511	23.52941176	3.107658158	13.31853496			
180512*	36.62597114	25.52719201	31.07658158			
180513	18.75693674	17.75804661	18.25749168			
180514	28.41287458	19.08990011	23.75138735			
180515	26.30410655	16.53718091	21.42064373			
180516	1.66481687	-0.776914539	0.443951165			
180517	45.61598224	33.74028857	39.67813541			
180518*	33.74028857	25.52719201	29.63374029			
180519*	35.9600444	-2.774694784	16.59267481			
180520	11.76470588	6.659267481	9.211986681			
180521	11.76470588	17.9800222	14.87236404			
180522	26.63706992	12.8745838	19.75582686			
180523	25.19422863	8.879023307	17.03662597			
180524	13.98446171	-34.07325194	-10.04439512			
180525*	31.85349612	29.30077691	30.57713651			
180526	27.52497225	23.0854606	25.30521643			
Neg	-8.2131	8.213097	0	-2.010968921	2.010968921	0
Pos	58.37958	60.48835	59.43396	63.07129799	53.93053016	58.50091408

* Represents samples that did not have congruent replicate samples

Table S4. Histological findings from 26 wild pigs and one fetus via light microscopy.

Sample ID	Heart	Tongue	Liver	Kidney	Spleen
180501	Rare, minimal lymphocytic inflammation	MF mild LP superficial inflammation; rare S	Diffuse vac; rare small clusters of neut	Minimal, MF LP in cortex	WNL
180502	WNL	WNL	Diffuse vac; focal IC necrosis with eos, LP, hist (mild)	WNL	--
180503	WNL	WNL	Diffuse vac	WNL	Macs
180504	WNL	WNL	Diffuse vac	WNL	Macs
180505	WNL	WNL	Diffuse vac	WNL	Macs
180506	WNL	WNL	Diffuse vac; rare small clusters of neut	WNL	Macs
180507	WNL	WNL	Diffuse vac	WNL	Macs
180508	WNL	WNL	Diffuse vac	WNL	Macs
180509	MF mild myocardial fibrosis with fiber loss (right heart)	WNL	Diffuse vac	WNL	Macs
180510	Medial hypertrophy of medium arteries (MF)	WNL	Diffuse vac	Focal lymphocytes in cortex, mild	Macs
180511	WNL	Many S	Diffuse vac	Focal lymphocytes in cortex, mild	Macs
180511F[a]	WNL	--	--	WNL	--
180512	Medial hypertrophy of medium arteries (MF)	Few S	WNL	WNL	Macs
180513	WNL	Few S	Diffuse vac; focal IC necrosis with eos, LP, hist (mild)	Rare lymphocytes in cortex, mild	Macs
180514	WNL	Few S	Diffuse vac	WNL	Macs
180515	Medial hypertrophy of medium arteries (MF); Rare S	Few S	Diffuse vac	WNL	Macs
180516	WNL	Rare S; MF (2) small foci of cartilagenous metaplasia	Diffuse vac	WNL	Macs
180517	WNL	Few S	Diffuse vac	WNL	Macs
180518	Rare S	Rare S; Focal, mild LP inflammation	Diffuse vac	WNL	Macs
180519	Medial hypertrophy of medium arteries (MF)	Focal, mild LP inflammation	Diffuse vac	Focal lymphocytes in cortex, mild	Macs
180520	Focal lymphocytes in myocardium	Rare S; MF (2) small foci of cartilagenous metaplasia	Diffuse vac	WNL	Macs
180521	WNL	Few S	Diffuse vac	Minimal, MF LP in cortex	WNL
180522	Medial hypertrophy of medium arteries (MF)	WNL	Diffuse vac	WNL	Macs
180523	WNL	WNL	Diffuse vac	Minimal, MF LP in cortex	Macs
180524	Focal lymphocytes in epicardium; medial hypertrophy of medium arteries (MF)	WNL	Diffuse vac	WNL	Macs
180525	WNL	WNL	Diffuse vac	WNL	Macs
180526	Medial hypertrophy of medium arteries (MF); focal, mild lymphohist inflammation	WNL	Diffuse vac	WNL	Macs

WNL = within normal limits; MF = multifocal; LP = lymphoplasmacytic; S = Sarcocysts; IC = indiviual cell; Vac = vacuolar change consistent with glycogen and/or lipid; Macs = macrophages (forming granulomatous reaction); Eos = eosinophil; Hist = histocytes; Neut = neutrophils

[a] Fetal tissue was sampled from this sow
